# The clinical importance of a cytokine network in the acute phase of sepsis

**DOI:** 10.1038/s41598-018-32275-8

**Published:** 2018-09-18

**Authors:** Hisatake Matsumoto, Hiroshi Ogura, Kentaro Shimizu, Mitsunori Ikeda, Tomoya Hirose, Hiroshi Matsuura, Sujin Kang, Kanae Takahashi, Toshio Tanaka, Takeshi Shimazu

**Affiliations:** 1 Department of Traumatology and Acute Critical Medicine, Osaka University Graduate School of Medicine, 2-15 Yamadaoka, Suita, Osaka 565-0871 Japan; 2 Department of Clinical Application of Biologics, Osaka University Graduate School of Medicine, Osaka University, 2-15 Yamadaoka, Suita, Osaka 565-0871 Japan; 3 Department of Medical Statistics, Osaka City University Graduate School of Medicine, 1-4-3 Asahi-machi, Abeno-ku, Osaka 545-8585 Japan

## Abstract

Sepsis remains a major cause of death. Cytokines interact closely with each other and play a crucial role in the progression of sepsis. We focussed on the associations of a cytokine network with prognosis and disease severities in sepsis. This retrospective study included 31 patients with sepsis and 13 healthy controls. Blood samples were collected from patients on days 1, 2, 4, 6, 8, 11 and 15 and from healthy controls. Levels of PAI-1, IFN-α, IFN-γ, IL-1β, IL-6, IL-8, IL-12/IL-23p40, IL-17A, TNF-α, MCP-1, IL-4 and IL-10 were measured. SOFA, JAAM DIC and ISTH DIC scores were evaluated at the same times blood samples were taken. Network analysis revealed a network formed by PAI-1, IL-6, IL-8, MCP-1 and IL-10 on days 1, 2 and 4 throughout the acute phase of sepsis. There were positive correlations of each cytokine and the combined score (IL-6 + IL-8 + IL-10 + MCP-1) with the SOFA, JAAM DIC and ISTH DIC scores throughout the acute phase. A Cox proportional hazards model focussed on the acute phase showed that the above combined score was significantly related with patient prognosis, suggesting that the cytokine network of IL-6, IL-8, MCP-1 and IL-10 could play a pivotal role in the acute phase of sepsis.

## Introduction

Sepsis remains one of the major causes of death in intensive care unit (ICU) patients. More than one in four people die of sepsis in the world each year^1^. Pathogen-associated molecular patterns and damage-associated molecular patterns bind to pattern recognition receptors on particular immune cells in the pathogenesis of sepsis. The activated immune cells induce an acute inflammatory response, which is mainly driven by not only pro-inflammatory cytokines but also anti-inflammatory cytokines that regulate the inflammatory response. An excess of pro-inflammatory cytokines can lead to endothelial injury and systemic inflammatory response syndrome, and severe cases can progress to disseminated intravascular coagulation (DIC) and multiple organ failure that eventually lead to death^2^.

Cytokine is the general term used to describe small protein mediators produced by immune cells. In general, cytokines include the interleukins (IL), interferons (IFN), chemokines and tumour necrosis factors (TNF). Cytokines can act on several cells and play a variety of roles such as in inflammation and cellular differentiation. It has been shown that cytokines enhance or suppress the production of other cytokines^3–5^. Therefore, it is important to focus on cytokine networks to reveal the complicated inflammatory pathogenesis in sepsis. Cytokine networks in chronic inflammatory diseases such as rheumatoid arthritis have already been clarified, and effective cytokine-targeted therapies have been developed^6^.

So far, it has been reported that several cytokines levels are related to the prognosis and severities of acute inflammatory diseases including sepsis^7–9^. However, no link between cytokine networks and the prognosis and severities of sepsis has yet been clarified. Cytokine profiling techniques^10–13^ have made it possible to evaluate the levels of multiple cytokines and clarify the status of cytokine networks. Therefore, the purpose of this study was to evaluate the relation of a cytokine network with patient prognosis and disease severities in sepsis.

## Patients and Methods

### Patients

This study was conducted with data obtained at the Department of Traumatology and Acute Critical Medicine, Osaka University Graduate School of Medicine from February 2014 to July 2015. The inclusion criteria were patients >18 years old with severe sepsis defined by the Sepsis-2 criteria^14^; however, all patients enroled in this study also retrospectively satisfied the Sepsis-3 criteria^15^. Patients who suffered cardiac arrest or were taking corticosteroids or immunosuppressive medications prior to arrival or the data related to those medications in the ICU were excluded. Healthy people with no previous history of chronic diseases were recruited as controls via public poster advertisements and selected randomly to match the age and gender of the patients.

This study followed the principles of the Declaration of Helsinki and was approved by the institutional review board of Osaka University Hospital (Permit Number: 16109). Informed consent was obtained from the patients or their relatives and the healthy volunteers for the collection of all blood samples.

### Blood samples

Blood samples were collected from patients until ICU discharge or death on days 1 (within 24 hours of the diagnosis), 2, 4, 6, 8, 11 and 15 (totalling a maximum of 7 samples collected per patient) and from the healthy controls. Separated serum and plasma samples for measuring cytokine concentrations were stored at −40 °C until analysis.

### Analyses of 11 cytokines

Serum concentrations of pro-inflammatory cytokines (IL-1β, TNF-α, IFN-α, IFN-γ, IL-6, IL-8, IL-12/IL-23p40, IL-17A and MCP-1) and anti-inflammatory cytokines (IL-4 and IL-10) were measured with the use of a cytometric bead array kit (BD Biosciences) by a FACSCanto II flow cytometer (BD Biosciences). The detectable range of each cytokine was 9.77 to 3000 pg/mL.

### Analyses of plasma levels of PAI-1

Plasma concentrations of plasminogen activator inhibitor-1 (PAI-1) were measured with an enzyme-linked immunosorbent assay kit (R&D Systems, Minneapolis, MN, USA). Frozen samples were thawed, and subsequent measurement processes were conducted according to the manufacturer’s protocol. Absorbance was analysed using a microplate reader (SH-9000Lab; Corona Electric Co., Ltd., Ibaraki, Japan). The minimum detectable level was less than 0.014 ng/mL.

### Severities and outcome assessment

The Acute Physiology and Chronic Health Evaluation (APACHE) II score was assessed at the enrolment of the patients with sepsis. The APACHE II score is designed to assess the severity of critically ill patients based on physiologic measurements, age and previous health status and is used for the prediction of outcome in critically ill patients^16^. The Sequential Organ Failure Assessment (SOFA) score was assessed at the same time points that blood samples were taken. The SOFA is a scoring system composed of six organ systems (comprising the respiratory, coagulation, hepatic, cardiovascular, renal and neurologic systems) and can be used for the evaluation of organ failure and prognosis^17^. DIC is a life-threatening complication that occurs based on an imbalance of the coagulation cascade. The severity of DIC was also evaluated at the same time points that blood samples were taken with two definitions of DIC: the Japanese Association for Acute Medicine (JAAM) DIC score^18^ and the International Society of Thrombosis and Haemostasis (ISTH) overt DIC score^19^. The JAAM score is sensitive for detecting septic DIC, and the ISTH overt DIC score is specific for diagnosing DIC. Both DIC scores correlate with each other^20^. The outcome measure was death within 28 days of diagnosis. Sepsis was divided into two phases based on a previous report: the acute phase (days 1–4) and the later phase (days 5–14)^21^.

### Statistical analysis

The levels of the 11 cytokines and of PAI-1 were transformed to common logarithm values to normalise the distribution of the data before the following analyses. The Dunnet test was used to evaluate the differences in each cytokine between the patients with sepsis and the healthy controls. The patients were respectively divided into two groups based on a previous report^22^ on day 1, day 2 and day 4: critical ill patients with sepsis (SOFA score on each day ≥12) and non-critical ill patients (SOFA score on each day <12). The Dunnet test was also used to assess differences between critical ill patients, non-critical ill patients and the healthy controls. Correlations between the 11 cytokines and PAI-1 were assessed by Ward’s hierarchical clustering analysis based on Pearson correlation coefficients. Network analysis was performed with Cytoscape^®^ software (www.cytoscape.org) version 3.5.1^23^. The network was based on the significant Pearson correlation coefficients between the 11 cytokines and PAI-1. The log2 fold changes were calculated by dividing average cytokine levels in the septic patients by the average levels in the healthy controls. The cytokines and PAI-1 with log2 fold change >1.5 were considered differentially increased. The network with major impact was visualised on the basis of the significant Pearson correlation coefficients between the cytokines and PAI-1. The combined score was calculated with the use of IL-1β, IL-6, IL-8, IL-10, MCP-1 and PAI-1, which are included in the networks with major impact, on the basis of a previous report^24^. Patients were divided into two groups based on the 75th percentile of each mediator’s levels. The patients with mediator levels greater than or equal to the 75th percentile value were assigned the value “1”, and those with mediator levels below the 75th percentile value were assigned the value “0”. The combined scores were calculated by adding each of the mediators’ values (i.e. the combination of three mediator scores consists of the individual value 0 or 1 or 2 or 3, the combination of four mediator scores consists of the individual value 0 or 1 or 2 or 3 or 4, the combination of five mediator scores consists of the individual value 0 or 1 or 2 or 3 or 4 or 5, and the combination of six mediator scores consists of the individual value 0 or 1 or 2 or 3 or 4 or 5 or 6). The combined scores A, B, C and D were calculated using combined mediator scores of (IL-1β, IL-6, IL-8, IL-10, MCP-1, PAI-1), (IL-6, IL-8, IL-10, MCP-1, PAI-1), (IL-6, IL-8, IL-10, MCP-1) and (IL-6, IL-8, MCP-1), respectively. Correlations of the 11 cytokines and PAI-1 and the combined scores with the SOFA and DIC scores were assessed by Spearman’s correlation coefficients. The strengths of the associations were divided into four groups based on the correlation coefficients: strong (>0.8), moderate (0.5 to 0.8), weak (0.3 to 0.5) and very weak (0.1 to 0.3).

Cox proportional hazards analysis with time-dependent covariates was performed based on the 11 cytokines and PAI-1 and the combined scores measured from day 1 to day 4 to evaluate the association of each value in the acute phase with death. It was reported that the maximum cytokine values and rapid increase of PAI-1 with a peak in the acute phase could reflect the severity of sepsis^25,26^. Therefore, the maximum values from three days (day 1, day 2 and day 4) were used for the analysis as time-dependent covariates to reflect the effect of the maximum values in the acute phase (i.e. day 1: the cytokine values measured on day 1; day 2: the maximum values from day 1 or day 2; and day 4: the maximum values from day 1, day 2 or day 4). The hazard ratio is provided as Q1 to Q3 to allow comparison of the strength of the association between the cytokines. We selected SOFA score and APACHE II score as confounders based on their strong relation with outcome in critically ill patients^16,17^. Competing risks were not taken into account because we focussed on overall survival.

To explore new predictive factors of sepsis, receiver operating characteristic (ROC) analysis with penalised maximum likelihood estimations were used to evaluate the associations of outcome with the SOFA score and several cytokines and PAI-1 and the combined scores while controlling for model overfitting. The penalised maximum likelihood estimation is one of the shrinkage approaches, which is really a special case of Bayesian modelling with a Gaussian prior^27^. A *P* value of <0.05 was considered to indicate statistical significance. Statistical analyses were performed with JMP Pro 13.0 for Windows (SAS Institute Inc., Cary, NC, USA) and R version 3.3.1 (R Foundation for Statistical Computing, Vienna, Austria).

## Results

### Patient characteristics

Thirty-one patients with sepsis and 13 healthy controls were consecutively enroled in this study (Table 1). In total, 156 blood samples collected from the 31 patients and 13 samples collected from the controls were analysed. At the time of enrolment, patients were in septic shock. Of the patients with sepsis, 23 were men and 8 were women, and the mortality rate of these patients was 22.5%. The median APACHE II, SOFA, ISTH DIC and JAAM DIC scores were 21.0 (16.0–29.0), 9.0 (5.0–11.0), 3.0 (2.0–4.0) and 4.0 (2.0–5.0), respectively. The sources of infection and comorbidities of the patients are shown in Table 1. There was no significant difference in age and sex between the patients with sepsis and the controls.

**Table 1:** Patient characteristics.

Characteristic	Sepsis (n = 31)	Controls (n = 13)
Age (years)	73.0 (65.0–81.0)	70.0 (45.0–73.0)
Sex, male/female	23/8	8/5
28-day mortality, n (%)	7 (22.5)	
APACHE II score	21.0 (16.0–29.0)	
SOFA score	9.0 (5.0–11.0)	
ISTH score	3.0 (2.0–4.0)	
JAAM score	4.0 (2.0–5.0)	
**Diagnosis at admission**		
Sepsis	11	
Septic shock	20	
**Site of infection**		
Chest	10	
Abdomen	11	
Soft tissue	7	
Urinary	1	
Others	2	
Comorbidity		
Diabetes mellitus	6	
Chronic kidney disease	2	
Connective tissue disease	3	
Solid tumour without metastasis	3	
Cerebrovascular disease	2	
Myocardial infarction	1	
Peptic ulcer disease	1	
Leukaemia	1	
Liver disease	1	

Note: Data are shown as group number or median (interquartile range). APACHE: Acute Physiology and Chronic Health Evaluation; SOFA: Sequential Organ Failure Assessment; ISTH: The International Society of Thrombosis and Haemostasis; JAAM: Japanese Association for Acute Medicine.

### Changes of 11 cytokines, PAI-1 levels and SOFA and DIC scores

**Figure 1:**
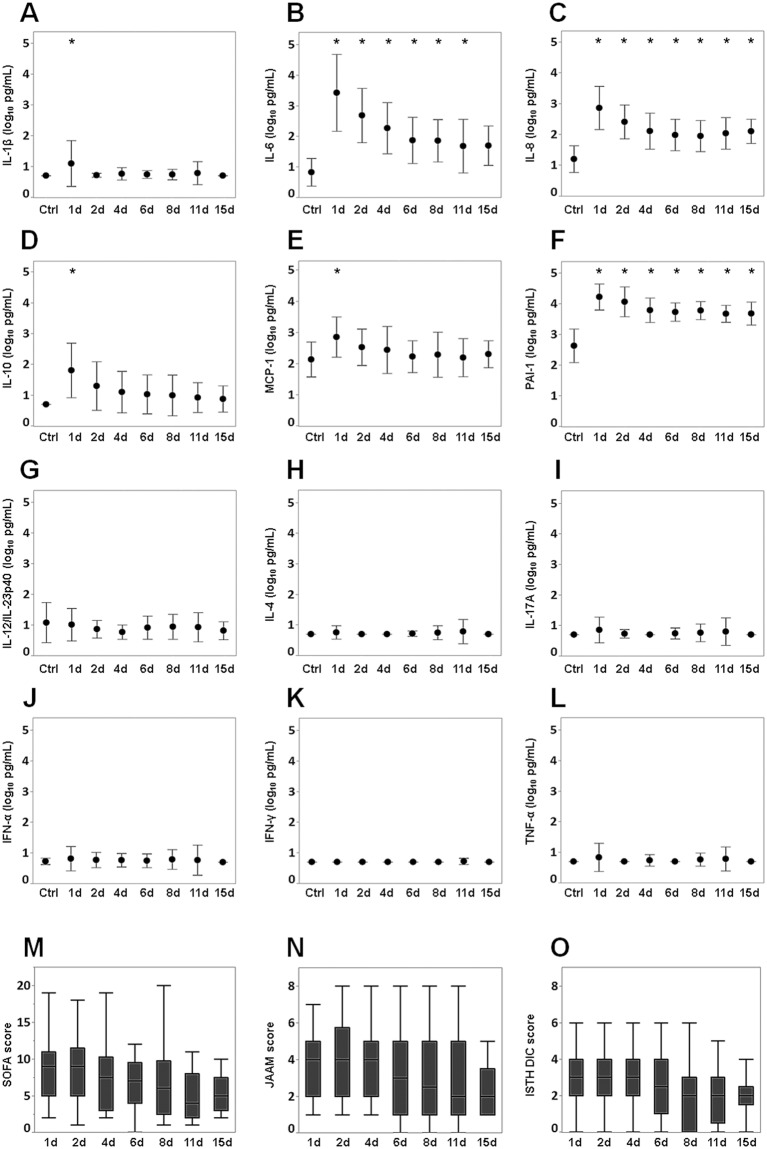
Changes in the levels of 11 cytokines and PAI-1 and in SOFA and DIC scores. (a) (**A**) Interleukin-1 beta (IL-1β), (**B**) interleukin-6 (IL-6), (**C**) interleukin-8 (IL-8), (**D**) interleukin-10 (IL-10), (**E**) monocyte chemotactic protein-1 (MCP-1), (**F**) plasminogen activator inhibitor-1 (PAI-1), (**G**) interleukin-12/23p40 (IL-12/IL-23p40), (**H**) interleukin-4 (IL-4), (**I**) interleukin-17A (IL-17A), (**J**) interferon-α (IFN-α), (**K**) interferon-γ (IFN-γ), and (**L**) tumour necrosis factor-α (TNF-α). The cytokine and PAI-1 values were transformed to common logarithm values to normalise the distribution of the data. All data are expressed as mean ± SD. Asterisks indicate a significant difference in cytokine and PAI-1 levels between control and septic patients on each day (*P* < 0.05). (b) Changes of (**M**) SOFA score, (**N**) JAAM score (**O**) and ISTH DIC score. The boxes indicate the lower and upper quartiles, the central line is the median, and the ends of the whiskers represent the maximum and minimum values. SOFA: Sequential Organ Failure Assessment; JAAM: Japanese Association for Acute Medicine; ISTH: International Society of Thrombosis and Haemostasis.

IL-8 and PAI-1 levels were significantly increased compared with those of the controls throughout the study period. Levels of IL-6 (days 1, 2, 4, 6, 8 and 11) and IL-1β, MCP-1and IL-10 (days 1) increased significantly compared with those of the controls. There were no significant differences in the levels of IFN-α, IFN-γ, IL-12/IL-23p40, IL-17A, TNF-α and IL-4 between the patients with sepsis and the controls (Fig. 1A). The levels of IL-6, IL-8, MCP-1, IL-10 and PAI-1 peaked on day 1 and then gradually decreased. A similar tendency was seen in the SOFA, JAAM and ISTH DIC scores (Fig. 1B).

### Cytokine (IL-1β, IL-6, IL-8, IL-10, MCP-1) and PAI-1 levels in critical and non-critical ill patients

**Figure 2:**
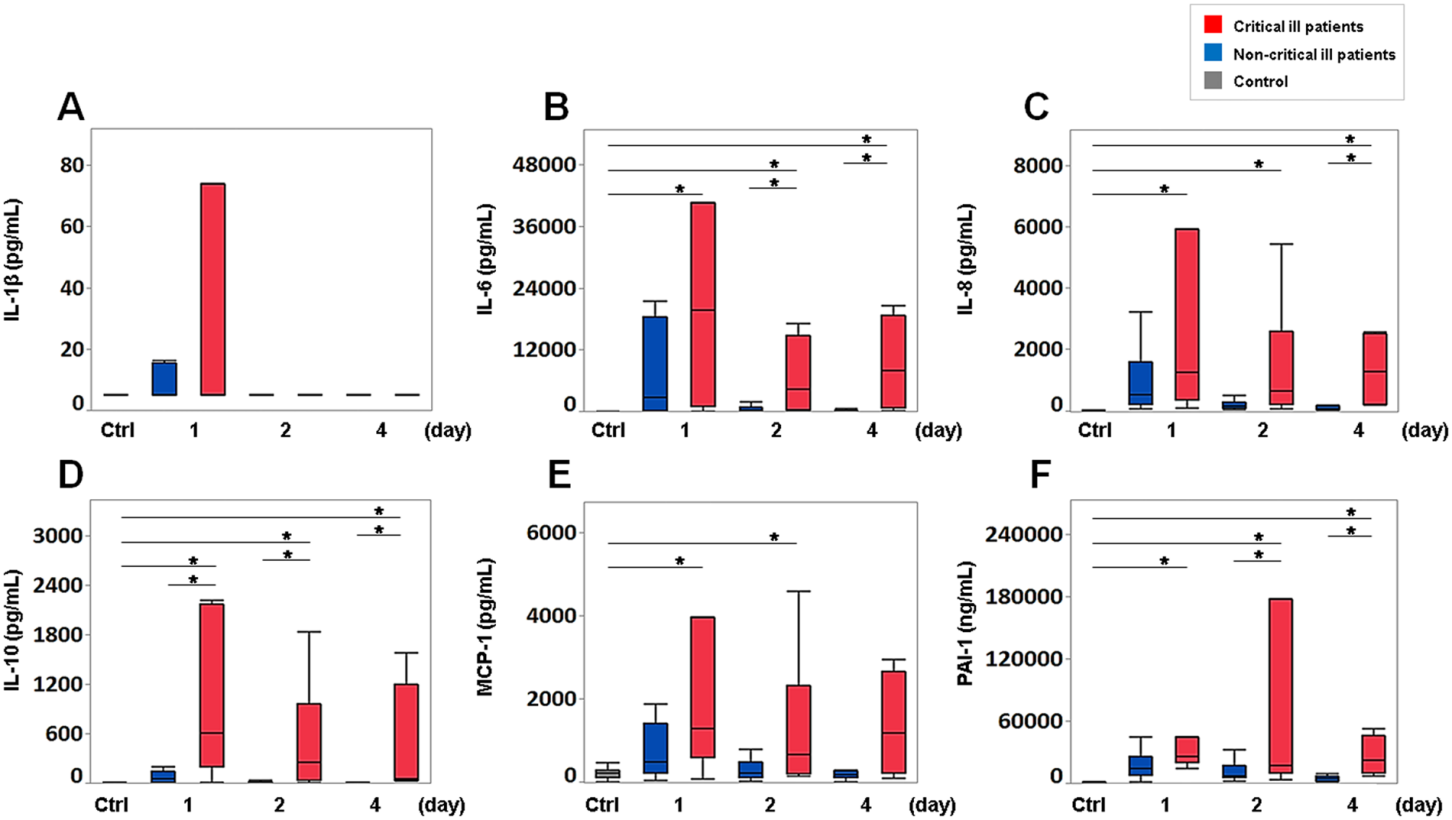
Levels of cytokines and PAI-1 in critical ill patients and non-critical ill patients. (**A**) Interleukin-1 beta (IL-1β), (**B**) interleukin-6 (IL-6), (**C**) interleukin-8 (IL-8), (**D**) interleukin-10 (IL-10), (**E**) monocyte chemotactic protein-1 (MCP-1), and (**F**) plasminogen activator inhibitor-1 (PAI-1) levels on day 1 (critically ill patients and non-critically ill patients: n = 7 and n = 24), day 2 (n = 6 and n = 18) and day 4 (n = 4 and n = 18), respectively. The boxes indicate the lower and upper quartiles, the central line is the median, and the ends of the whiskers represent the maximum and minimum values. Asterisks indicate a significant difference between critically ill patients, non-critically ill patients and controls (*P* < 0.05).

We evaluated the levels of cytokines IL-1β, IL-6, IL-8, MCP-1 and IL-10 and of PAI-1, which increased over the acute phase (Figs 1 and 2), in the critical and non-critical ill patients. The levels of IL-10 (days 1, 2 and 4) and IL-6 and PAI-1 (days 2 and 4) and IL-8 (day 4) in the critically ill patients were significantly increased compared to those in the non-critically ill patients. The levels of IL-6, IL-8, IL-10 and PAI-1 in the critically ill patients were significantly increased compared over the acute phase. MCP-1 levels in the critically ill patients showed significant increases compared with those of the controls on days 1 and 2. IL-1β levels showed no significant difference between the critically and non-critically ill patients and the controls in the acute phase (Fig. 2).

### Hierarchical clustering and network visualisation among 11 cytokines and PAI-1

**Figure 3:**
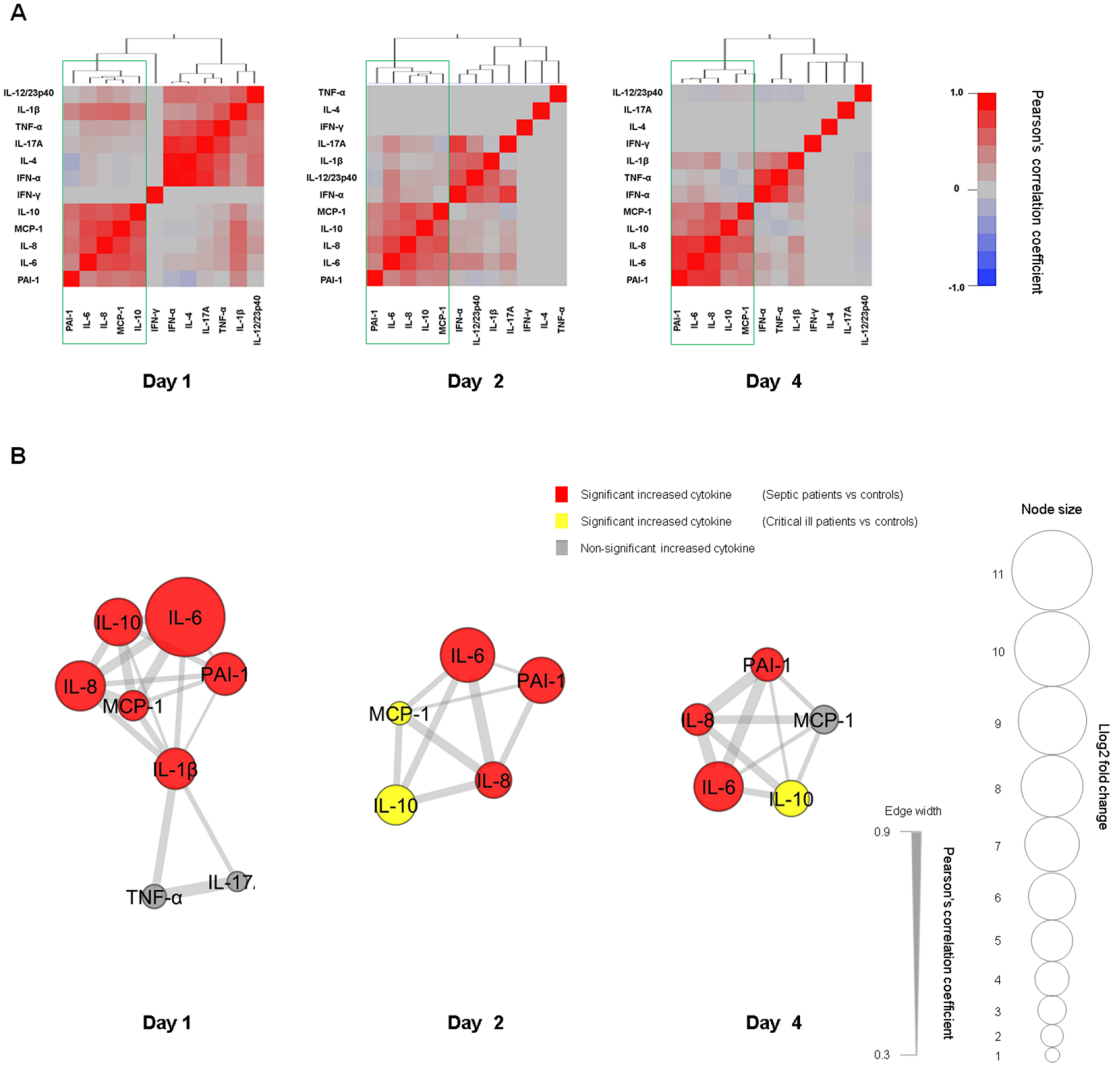
Hierarchical clustering and network visualisation. The cytokine and PAI-1 values were transformed to common logarithm values to normalise the distribution of the data. (**A**) Hierarchical clustering of Pearson’s correlations between cytokines and PAI-1. The outlined boxes show the common cytokine network in the acute phase of sepsis (days 1–4). (**B**) This network visualises the significant correlations of (**A**). The cytokines and PAI-1 with log2 fold change (i.e. average cytokine levels in sepsis patients/average cytokine levels in controls) > 1.5 were included. The size of each node was determined based on the log2 fold change. Red, yellow, and grey colours indicate a significant increase in the cytokines and PAI-1 levels between septic patients and controls, between critically ill patients and controls, and no difference between septic or critically ill patients and controls, respectively. Increased cytokines compared to those of the controls and the connections are shown to represent networks with major impact. IFN-α: interferon-α; IFN-γ: interferon-γ; IL-1β: interleukin-1 beta; IL-6: interleukin-6; IL-8: interleukin-8; IL-12/IL-23p40: interleukin-12/23p40; IL-17A: interleukin-17A; TNF-α: tumour necrosis factor-α; MCP-1: monocyte chemotactic protein-1; IL-4: interleukin-4; IL-10: interleukin-10; PAI-1: plasminogen activator inhibitor-1.

We assessed the hierarchical clustering of Pearson’s correlations among the 11 cytokines and PAI-1 during the study period to reveal networks. The common cluster composed of IL-6, IL-8, MCP-1, IL-10 and PAI-1 was formed on days 1, 2 and 4 (Fig. 3A). The networks comprised of IL-1β, IL-6, IL-8, MCP-1, IL-10 and PAI-1 were seen on day 1, and the common networks formed by IL-6, IL-8, MCP-1, IL-10 and PAI-1 were seen on days 1, 2 and 4 (Fig. 3B).

### Spearman’s correlations between 11 cytokines and PAI-1 and combined scores and SOFA and DIC scores

**Figure 4:**
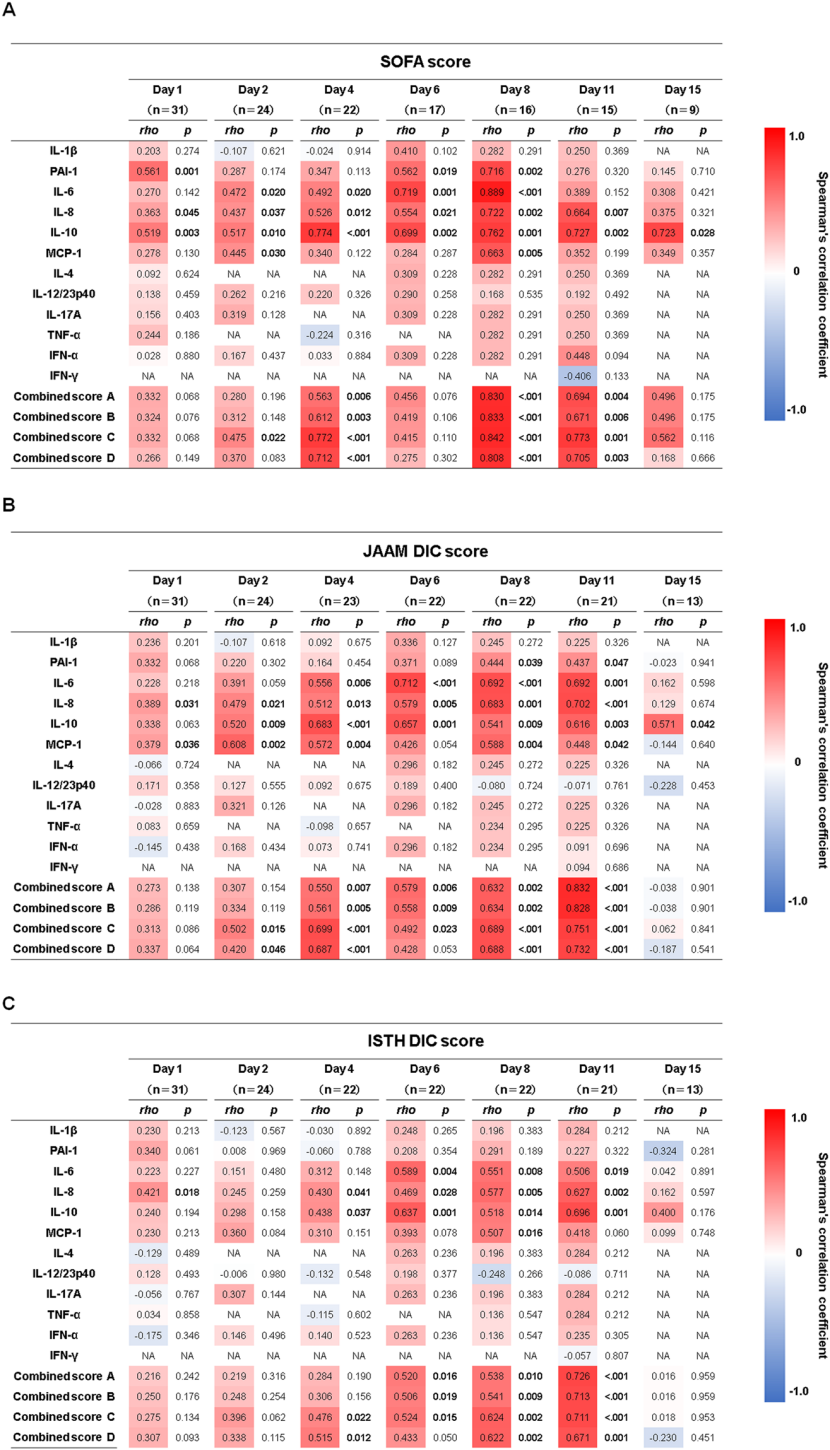
Correlations between cytokines and PAI-1 and the combined scores and disease severities in the patients with sepsis. Correlations with (**A**) SOFA score, (**B**) JAAM score and (**C**) ISTH score. The red colour indicates a positive correlation, and the blue colour indicates a negative correlation. The *P* values in bold font indicate statistical significance. The levels of the 11 cytokines and PAI-1, which were transformed to common logarithm values, and the combined scores were used for analysis. SOFA: Sequential Organ Failure Assessment; JAAM: Japanese Association for Acute Medicine; ISTH: International Society of Thrombosis and Haemostasis; PAI-1: plasminogen activator inhibitor-1; IL-1β: interleukin-1 beta; IL-6: interleukin-6; IL-8: interleukin-8; IL-10: interleukin-10; MCP-1: monocyte chemotactic protein-1; IL-4: interleukin-4; IL-12/IL-23p40: interleukin-12/23p40; IL-17A: interleukin-17A; TNF-α: tumour necrosis factor-α; IFN-α: interferon-α; IFN-γ: interferon-γ; NA: not available. Combined scores A, B, C and D: combined scores of (IL-1β, IL-6, IL-8, IL-10, MCP-1, PAI-1), (IL-6, IL-8, IL-10, MCP-1, PAI-1), (IL-6, IL-8, IL-10, MCP-1) and (IL-6, IL-8, MCP-1), respectively.

We chronologically investigated Spearman’s correlation coefficients between the 11 cytokines and PAI-1 and the combined scores. There were very weak to strong positive correlations of the SOFA score with IL-6, IL-8, MCP-1, IL-10 and PAI-1 and combined scores A, B, C and D throughout the study period (Fig. 4A). Very weak to strong positive correlations were seen between the JAAM DIC scores and IL-6, IL-8, MCP-1, IL-10 and PAI-1 and combined scores A, B, C and D and between the ISTH DIC and IL-6, IL-8, MCP-1, IL-10 and combined scores A, B, C and D on day 1 through day 11 (Fig. 4B,C).

### Cox proportional hazards analysis with time-dependent covariates for survival

**Figure 5:**
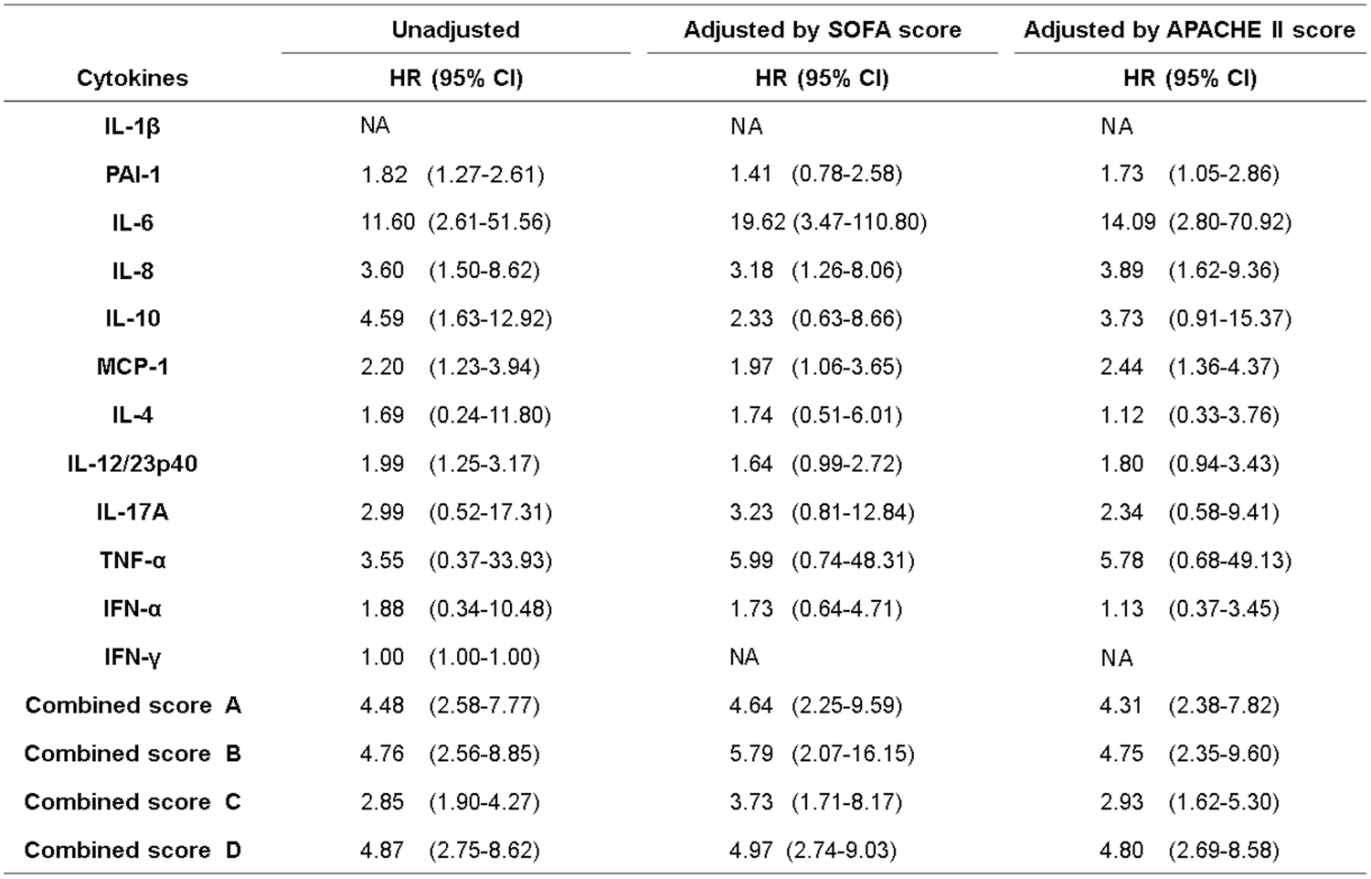
Cox proportional-hazards analysis with time-dependent covariates for survival in the patients with sepsis. The cytokine and PAI-1 values and the combined scores were measured during the acute phase (day 1, day 2 and day 4). The cytokine and PAI-1 values were transformed to common logarithm values to normalise the distribution of the data. The maximum values from day 1; those from day 2, i.e. the maximum values from day 1 or day 2; and those from day 4, i.e. the maximum values measured on day 1, day 2 or day 4, were used as time-dependent covariates. The hazard ratio is provided as Q1 to Q3. The values were adjusted by APACHE II score and SOFA score. APACHE: Acute Physiology and Chronic Health Evaluation; SOFA: Sequential Organ Failure Assessment; PAI-1: plasminogen activator inhibitor-1; IL-1β: interleukin-1 beta; IL-6: interleukin-6; IL-8: interleukin-8; IL-10: interleukin-10; MCP-1: monocyte chemotactic protein-1; IL-4: interleukin-4; IL-12/IL-23p40: interleukin-12/23p40; IL-17A: interleukin-17A; TNF-α: tumour necrosis factor-α; IFN-α: interferon-α; IFN-γ: interferon-γ; NA: not available. Combined scores A, B, C and D: combined scores of (IL-1β, IL-6, IL-8, IL-10, MCP-1, PAI-1), (IL-6, IL-8, IL-10, MCP-1, PAI-1), (IL-6, IL-8, IL-10, MCP-1) and (IL-6, IL-8, MCP-1), respectively.

To evaluate the relation between each cytokine and PAI-1 and the combined scores and patient prognosis, Cox proportional hazards analysis with time-dependent covariates was conducted. The cytokine and PAI-1 values were transformed to common logarithm values to normalise the distribution of the data before the analyses. The maximum value of each cytokine and PAI-1 and the combined scores measured on 3 days in the acute phase was used for the time-dependent covariate to highlight the acute phase. IL-6, IL-8, MCP-1, IL-10, PAI-1, IL-12/23p40 and combined scores A, B, C and D showed significant correlation with patient prognosis. IL-6, IL-8, MCP-1 and combined scores A, B, C and D adjusted by the SOFA score or APACHE II score showed significant associations with prognosis (Fig. 5).

### ROC analysis of the cytokines and PAI-1 and combined scores and SOFA score

To investigate potential prognostic biomarkers, ROC analysis was performed with IL-1β, IL-6, IL-8, MCP-1, IL-10 and PAI-1, which had increased in the acute phase, and the combined scores and the SOFA score on day 1. Assessment with the SOFA score is inevitable for the diagnosis of sepsis^15^ and is an important prognostic marker in the clinical setting. Therefore, we assessed whether the AUC analysed by the SOFA score with each cytokine increased compared with the AUC analysed by the SOFA score only.

**Figure 6:**
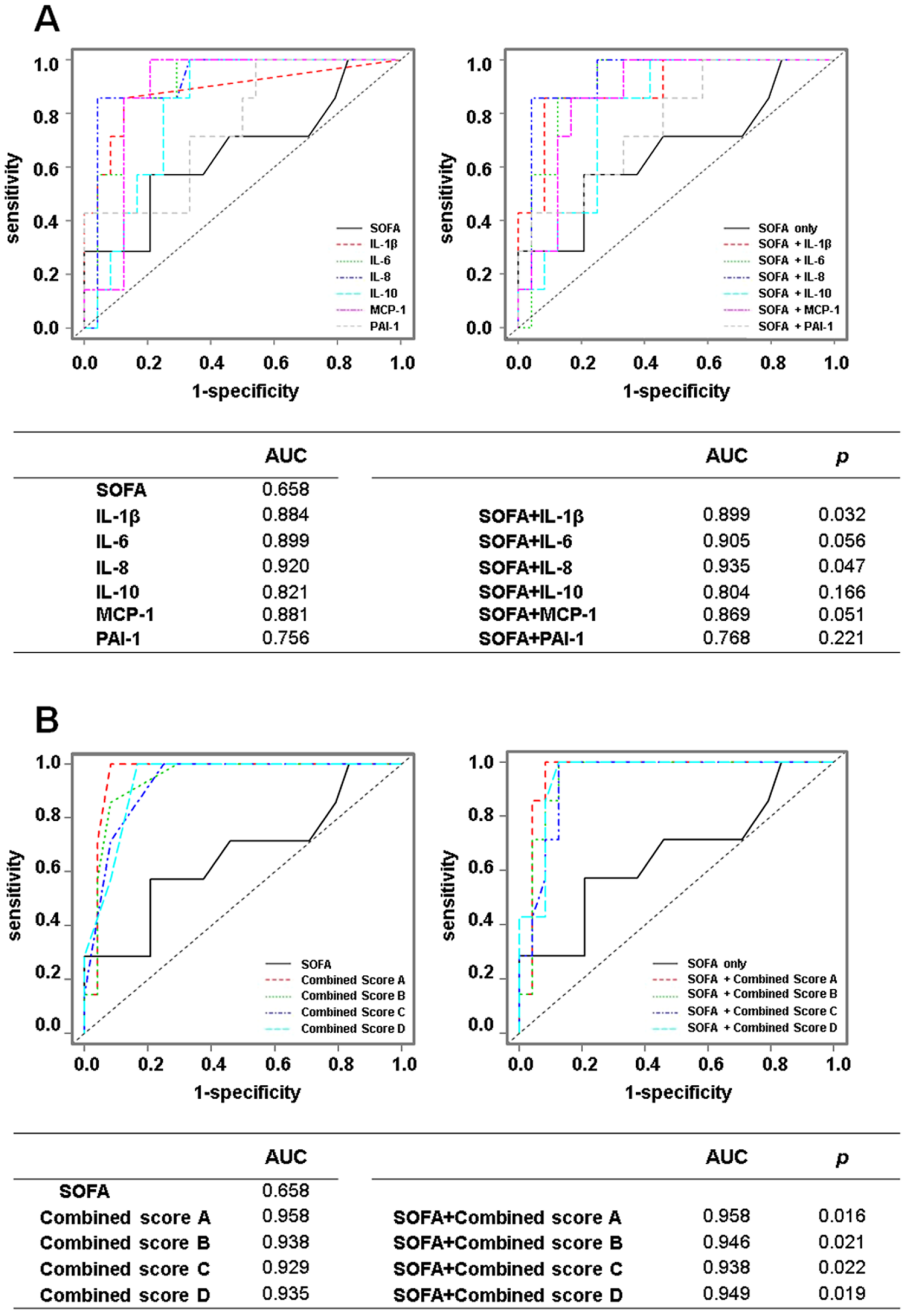
ROC analysis using the cytokines and PAI-1 and the SOFA score in the patients with sepsis. The levels of the 11 cytokines and PAI-1, which were transformed to common logarithm values, and the combined scores were used for analysis. The AUC was calculated to evaluate the prognostic accuracy of each marker on day 1 (**A,B**). The AUC analysed by the SOFA score with each cytokine was compared to the AUC analysed by the SOFA score only. The *P* values indicate statistical significance. AUC: area under the ROC curve; ROC: receiver operating characteristic; SOFA: Sequential Organ Failure Assessment; IL-1β: Interleukin-1 beta; IL-6: interleukin-6; IL-8: interleukin-8; IL-10: interleukin-10; MCP-1: monocyte chemotactic protein-1; PAI-1: plasminogen activator inhibitor-1. Combined scores A, B, C and D: combined scores of (IL-1β, IL-6, IL-8, IL-10, MCP-1, PAI-1), (IL-6, IL-8, IL-10, MCP-1, PAI-1), (IL-6, IL-8, IL-10, MCP-1) and (IL-6, IL-8, MCP-1), respectively.

The AUCs of IL-1β, IL-6, IL-8, MCP-1, IL-10, PAI-1, combined scores A, B, C and D and the SOFA score were 0.884, 0.899, 0.920, 0.881, 0.821, 0.756, 0.958, 0.938, 0.929, 0.935 and 0.658, respectively. The AUCs of the SOFA score with IL-1β (0.899), IL-8 (0.935), combined score A (0.958), combined score B (0.946), combined score C (0.938) and combined score D (0.949) were significantly increased compared with that of the SOFA score only (Fig. 6).

## Discussion

In this study, the levels of IL-1β, IL-6, IL-8, MCP-1 and PAI-1, which reflect endothelial injury, significantly increased in the early acute phase (day 1) of septic patients compared with those of the controls (Fig. 1A). The network analysis revealed a network of these mediators with weak to moderate correlations (Fig. 3B). A variety of cytokines are produced from immune cells in sepsis. IL-1β, which is mainly generated by activated monocytes and macrophages^28^, acts on different immune cells including endothelial cells. The activated immune cells then produce cytokines such as IL-6, IL-8 and MCP-1^29,30^. This suggests that IL-1β might promote the cytokine network formed by IL-6, IL-8 and MCP-1.

TNF-α is recognised as an important pro-inflammatory cytokine that produces cytokines such as IL-6, IL-8 and MCP-1 in addition to IL-1β^31,32^. In this study, TNF-α was not related to those cytokines (Fig. 3B), which indicates that the effect of TNF-α as a pro-inflammatory cytokine is limited.

This study showed significant increases of the IL-6, IL-8 and PAI-1 levels of the septic patients compared with those of the controls throughout the acute phase (Fig. 1A). The IL-10 levels of the critically ill patients showed significant increases compared with those of the non-critically ill patients and the controls throughout the acute phase. There were significant increases of MCP-1 levels on days 1 and 2 and on day 4 (t-test, P = 0.045) compared with those of the controls (Fig. 2). Hierarchical clustering analysis revealed a cluster formed by IL-6, IL-8, IL-10, MCP-1 and PAI-1, and network analysis clearly indicated a network of these mediators, which showed weak to strong correlations throughout the acute phase (Fig. 3A,B). Endothelial injury, which is strongly associated with the progression of sepsis^33^, can proliferate to generate cytokines such as IL-6, IL-8 and MCP-1. This indicates that endothelial cell injury might play a role in forming the cytokine network composed of IL-6, IL-8 and MCP-1 throughout the acute phase of sepsis. Also, the augmentation of IL-6, IL-8 and MCP-1 could proliferate the inflammation by endothelial cells as a positive feedback system^32,34,35^. Among these cytokine levels, the level of IL-6 increased most strikingly over the acute phase (Fig. 1A). The high concentration of IL-6 binds to the soluble form of IL-6 receptor (IL-6R), resulting in the IL-6/IL-6R complex. This complex combines with the signal transducing component glycoprotein 130 on the cells, including endothelial cells, to elicit IL-6 signal activation. This process accelerates endothelial injury and leads to the increasing generation of IL-6, IL-8 and MCP-1^32^.

The cytokine network in the acute phase was formed by the pro-inflammatory cytokines IL-6, IL-8 and MCP-1 and an anti-inflammatory cytokine, IL-10. Recently, the concurrent presence of both pro-inflammatory and anti-inflammatory mediators from the onset of sepsis has been described^36^. Our results were consistent with this report and suggest that both pro-inflammatory and anti-inflammatory responses correlate with the pathogenesis of sepsis in a mutual relationship^37^.

The pro-inflammatory cytokines are closely related with the progression of the coagulation process in sepsis. Excessive pro-inflammatory cytokines promote the expression of tissue factor, which is predominantly synthesised by activated monocytes^38,39^, causing coagulation disorder and microthrombi formation^2^. The formation of microthrombi could contribute to microcirculatory dysfunction and result in multiple organ failure that leads to death^40^. In our results, cytokines in the cytokine network (IL-6, IL-8, MCP-1 and IL-10) and the SOFA, JAAM and ISTH DIC scores showed similar time-dependent changes (Fig. 1A,B). Very weak to moderate positive correlations of each of the cytokines and combined score C (IL-6 + IL-8 + IL-10 + MCP-1) with the SOFA, JAAM DIC and ISTH DIC scores were seen over the acute phase (Fig. 4A–C). Also, in the Cox proportional hazards model, which focussed on the acute phase, combined score C (IL-6 + IL-8 + IL-10 + MCP-1) adjusted by SOFA or APACHE II scores showed a significant relation with the prognosis of the patient (Fig. 5). This suggests that the cytokine network composed by the pro-inflammatory cytokines IL-6, IL-8 and MCP-1, when interacting with endothelial cells, could facilitate the progression of sepsis based on the coagulation disorder, leading to a lethal outcome. However, the anti-inflammatory cytokine IL-10 might act as a negative feedback mechanism against the inflammatory response.

To discover clinically useful markers of prognosis on day 1, we compared the AUCs analysed by SOFA score with each cytokine in the cytokine network (IL-1β, IL-6, IL-8, MCP-1 and IL-10) and PAI-1 and the combine scores to the AUC of the SOFA score only. The AUC analysed by the SOFA score with combined score A (IL-1β + IL-6 + IL-8 + IL-10 + MCP-1 + PAI-1) (0.958) was highest and was significantly increased compared with the AUC of the SOFA score only (0.658). The increased AUC of the SOFA score with combined score A was the same as that of combined score A only (0.958) (Fig. 6B). This suggests that the combined scores of IL-1β + IL-6 + IL-8 + IL-10 + MCP-1 + PAI-1 could be a useful prognostic marker without using the SOFA score.

Limitations of this study are the relatively small number of patients included and the use of data from a single institution. Further study is necessary to clarify the role of cytokine networks in the pathogenesis of sepsis. We believe that these findings will have implications for the management of patients with sepsis.

## Conclusions

Cytokine profiles were assessed in patients with sepsis. We found that IL-6, IL-8, MCP-1 and IL-10 formed a cytokine network in the acute phase of sepsis and that the combined score of IL-6 + IL-8 + IL-10 + MCP-1 correlated with patient prognosis and disease severity.
